# 

**Published:** 1904-03-15

**Authors:** 


### ANNOUNCEMENTS.

THE Indiana State Dental Society will hold its annual meeting at Indianapolis, June 14th to 16th. It is hoped that this will be a good meeting, as efforts are being made to secure a large attendance and a good program.

THE Anderson (Indiana) Dental Society was organized January 25th, 1904, and elected the following officers:

President, C. W. Orland; Vice-President, H. E. Schellenger; Secretary and Treasurer, R. T. Brookins.

THE joint meeting of the Central Michigan and South-Western Michigan Dental Associations will be held in Grand Rapids, Tuesday and Wednesday, April 12th and 13th. Headquarters hotel Pantlind. This will be a large meeting and the program will be up to date. Everybody can come. C. W. Johnson, D.D.S., Sec'y Southwestern Association, Lawton, Mich; G. F. Smith, D.D.S., Sec'y Central Michigan Association, Belden, Mich.

MEMBERS of State Boards who desire the proceedings of the National Association of Examiners should write for them to the Secretary, Dr. Chas. A. Meeker, 29 Fulton St., Newark, N. J.

DR. J. E. Hinkins has been appointed chairman of the committee on organization for the Inetrnational Dental Congress for the State of Illinois, in place of Dr. F. H. Zinn. His address is 151 E. 53d street, Chicago.

THE Michigan State Board of Examiners meets May 10th, 1904, at Grand Rapids. W. C. McKinney, Secretary, Saginaw, Mich.

THE Minnesota State Dental Association meets June 16th to 18th, in St. Paul.

THE Eastern Indiana Dental Association will meet at Richmond, Ind., the first week in May.

